# Supplementary material for: Impact of the COVID-19 Pandemic on Epidemiological Trends in Pediatric Cervical Abscess-Forming Infections
Source: Microorganisms. 2025 Jan 17;13(1):190. doi: 10.3390/microorganisms13010190 (PMC11767812; doi:10.3390/microorganisms13010190)
Supplement: Supplementary file 1 [file microorganisms-13-00190-s001.zip › microorganisms-3382639-supplementary.pdf]

Supplementary Table (Table S1)

Table S1: Initial empiric intravenous antibiotic selected for each disease.

	superfical cervical	retropharyngeal	deep neck	peritonsillar
ABPC	4	1	1	2
ABPC/sbt	67	25	20	41
CEZ	16	3	2	4
CTX	1	3	3	2
MEPM	1	1		
others	4			2
unknown	3	1		

ABPC：ampicillin ABPC/sbt: ampicillin/sulbactam CEZ: cefazolin CTX: cefotaxime MEPM: meropenem
